# Retention of skull anatomy knowledge in dental education: a comparative study

**DOI:** 10.3389/fdmed.2025.1596610

**Published:** 2025-06-17

**Authors:** Noora Helene Thune, Anna Tostrup Kristensen, Qalbi Khan, Tor Paaske Utheim, Hugo Lewi Hammer, Amer Sehic

**Affiliations:** ^1^ Institute of Oral Biology, Faculty of Dentistry, University of Oslo, Oslo, Norway; ^2^ Department of Medical Biochemistry, Oslo University Hospital, Oslo, Norway; ^3^ Faculty of Social and Health Sciences, Inland Norway University of Applied Science, Elverum, Norway; ^4^ Department of Plastic and Reconstructive Surgery, Oslo University Hospital, Oslo, Norway; ^5^ Department of Computer Science, Faculty of Technology, Art and Design, Oslo Metropolitan University, Oslo, Norway

**Keywords:** anatomy, basic sciences, cranium, clinical dentistry, knowledge retention

## Abstract

There is limited evidence regarding the retention of essential basic science knowledge among dental students and professionals. Understanding the anatomy of the skull, osteology, is crucial as it provides the structural framework essential for comprehending head anatomy, where various soft tissue components are organized. This study aims to evaluate and compare the retention of osteological knowledge across different stages of dental education and professional practice. Specific anatomical structures on selected skull bones and the complete cranium, taught at the pre-clinical level and including clinically and radiographically significant landmarks, were marked for assessment. The study evaluated the ability of second year and fifth year undergraduate dental students, as well as postgraduate students at various stages of specialist training in different dental fields, to independently identify these marked anatomical structures. The study demonstrated significantly higher identification accuracy among second year students compared to fifth year and postgraduate students (*p* < 0.05). Second year students achieved over 90% accuracy for individual skull bones, with slightly lower accuracy for the entire cranium (85.9%). Fifth year students showed markedly lower retention, with accuracy below 50%, ranging from 20.8% (cranium) to 48.3% (mandible). Postgraduate students performed similarly to fifth year students, notably with only 11.8% accuracy for the sphenoid bone. Significant differences in accuracy were observed among postgraduate specialties (*p* < 0.05), with oral surgery and oral medicine specialists achieving the highest accuracy (81.2% for the mandible). However, no significant correlation between years of experience and accuracy was observed among the postgraduate groups (*p* = 0.45). Our results indicate that clinically and radiologically relevant anatomical knowledge is better retained over time, while overall osteological knowledge significantly declines. This highlights the need for strategies beyond initial learning to enhance long-term retention. Integrating clinical, radiological, and surface anatomy into continuing dental education could substantially improve knowledge retention. Furthermore, our findings suggest potential benefits from increased vertical integration and encourage broader discussion regarding the traditional separation between pre-clinical and clinical training phases.

## Introduction

Basic sciences form the foundation of both clinical dentistry and medicine, playing a crucial role in shaping education and practice (1, 2). This essential knowledge is important for effective communication with patients, accurate diagnoses, and treatment planning, with its clinical relevance aiding in long-term retention (3). However, there is limited data on the retention of basic science knowledge among dental students and professionals. Research highlights a significant decline in retention post-graduation, with studies indicating that medical graduates retain only 67%–75% of their knowledge within the first year, which drops to below 50% by the second year (4). Similar trends have been observed among undergraduate medical students, particularly as they progress into their clinical training years (4–6). Contributing factors include the limited emphasis on basic sciences in clinical textbooks and the overwhelming amount of clinical information that students must memorize (5, 6).

Anatomy is fundamental to preclinical dental education and a critical qualification for any dental professional. A thorough grasp of anatomy, particularly head and neck anatomy, underpins the ability of dental students to perform surgical and anesthetic procedures, as well as to conduct patient examinations (7). The inherent connection between dentistry and general medicine further underscores the necessity for a comprehensive understanding of human anatomy (8, 9), enabling dentists to effectively collaborate with specialists across other medical fields (10). Given this, anatomy departments bear the responsibility of laying a solid foundation to support clinical education and ensure the training of competent dentists. However, several controversies revolve around the optimal methods and extent to which anatomical sciences should be taught (11, 12), and it has been suggested that postgraduate education should also emphasize maintaining proficiency in specific anatomical knowledge (13). To effectively impart anatomical knowledge, it is essential to design an engaging and motivational curriculum, as motivation and learning are closely linked in academic settings (11). Studies have shown that focusing on clinically relevant topics enhances student motivation (14), and integrating anatomical instruction with surgical and radiological contexts improves both understanding and motivation (15). Additionally, the perceived relevance of basic medical sciences has been positively associated with knowledge retention, particularly within integrated, case-based learning environments (16). Considering these findings, it is likely that integrating clinical relevance into the anatomical curriculum could enhance both student motivation and knowledge retention, thereby improving educational outcomes for future dentists.

Understanding the anatomy of the skull, osteology, is a fundamental basis for comprehending the anatomy of the head, as the skull forms the framework around which the soft tissue components are structured. Therefore, the study of the osteology receives substantial emphasis in the curriculum to establish a solid basis for subsequent anatomical learning. At the Faculty of Dentistry, University of Oslo, the osteology component is introduced in the second year through a structured combination of lectures and practical sessions. Initially, students attend two 45 min lectures that provide a general overview of the human cranium, including its constituent bones, major anatomical features, and organization. Following the lectures, students participate in a total of 12 h of hands-on practical course, delivered over six sessions across a three-week period. During these sessions, students examine real human cranial bones, working individually or in small groups with bone boxes that contain a complete cranium as well as individual bones for focused study. To support their learning, students are provided with a comprehensive osteology compendium. This resource offers detailed descriptions of each cranial bone, accompanied by two-dimensional annotated illustrations. The annotations highlight specific anatomical structures, which students are expected to label in the compendium and identify on the real bones. While the instructional design does not incorporate supplemental digital resources such as videos or online image databases, students are encouraged to explore additional e-learning tools independently, both during class and in their own time. Assessment in osteology comprises both theoretical and practical components. The written examination requires students to describe a given bone, including its general features and detailed anatomical landmarks. In the practical examination, students are presented with real bones and tasked with identifying pre-marked anatomical structures.

The locations of muscles, blood vessels, nerves, and glands are largely described in relation to the anatomy of the skull. Furthermore, the terminology associated with these structures is frequently derived from osteological principles. Due to the skull's complex development and the involvement of critical anatomical structures, understanding its anatomy holds significant clinical and surgical relevance for dentists. Moreover, this knowledge is crucial for dental professionals, as many dentists routinely interpret various radiographic examinations, such as orthopantomograms (OPG) and cone-beam computed tomography (CBCT). Therefore, skull anatomy is regarded as an essential body of knowledge that must be preserved and reinforced throughout dental education and professional practice. To the best of our knowledge, the retention of osteological knowledge in dental education and profession has not been previously studied. The present study aims to assess and compare osteological knowledge among dental students in their second and fifth years of study, as well as among postgraduate students at varying stages of their careers, who are currently pursuing specialist training in different dental fields.

## Materials and methods

### Student cohorts

This study was conducted at the Faculty of Dentistry, University of Oslo, with the objective of comparing osteological knowledge among two cohorts of Master of Dentistry students and a group of postgraduate students undergoing specialist training in various dental fields. The first cohort consisted of 55 s year undergraduate students, who were assessed in their anatomy examination in 2022. This same cohort, now consisting of 30 students, was re-evaluated 2.5 years later, on identical osteological tasks during their fifth and final year in the program, to measure retention and progress in their osteological knowledge. The undergraduate student cohort had relatively homogeneous age and sociodemographic backgrounds, with most students aged between 20 and 25 years, reflecting the limited sociodemographic variation typical of the Norwegian population. However, gender distribution was skewed, with females comprising approximately 70% of this group.

The postgraduate cohort included representatives from all seven specialist training programs offered at the Faculty of Dentistry, University of Oslo: endodontics, prosthodontics, pediatric dentistry, periodontology, orthodontics, maxillofacial radiology, and oral surgery and oral medicine. The group consisted of 36 postgraduate students, all of whom were qualified dentists who had completed their undergraduate dental education in various European countries: Norway (*n* = 13), Poland (*n* = 5), Sweden (*n* = 3), Latvia (*n* = 2), Serbia (*n* = 1), Lithuania (*n* = 1), Germany (*n* = 1), and Greece (*n* = 1). The participants had varying lengths of clinical experience and different time intervals since graduation, ranging from 2 to 22 years. The mean years of clinical experience per specialty group are presented in Table 2. By including participants from a diverse range of dental specialties, the study aimed to assess osteological knowledge across a broad spectrum of professional dental expertise.

### Selected skull bones with marked anatomical structures

**Figure 1:**
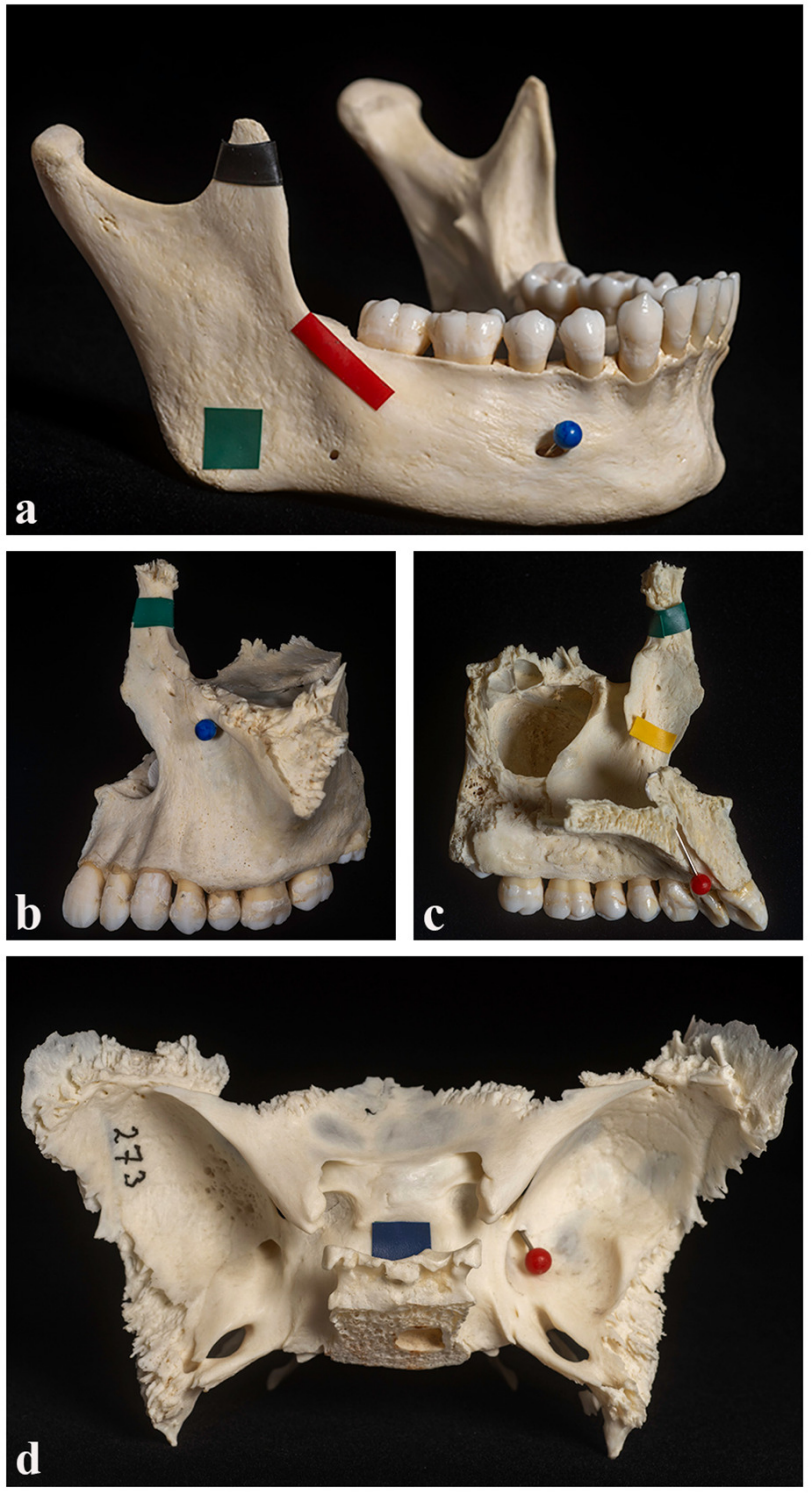
Examples of marked structures on selected bones. Three individual skull bones are shown as examples: the mandible **(a)**, the maxilla in lateral **(b)** and medial **(c)** views, and the sphenoid bone **(d)**. Each bone features precisely marked anatomical structures, highlighted using coloured electrical tape or pins with coloured heads for improved visibility.

Four individual skull bones, the mandible, maxilla, sphenoid, and temporal bone, along with a complete cranium, were selected as anatomical specimens for this study. Each of these five specimens featured four precisely marked anatomical structures, which were identified using coloured electrical tape or pins with coloured heads to ensure clear visibility (Figure 1). All anatomical structures on the cranial bones used in this study were marked by the same individual, the senior author of this manuscript, a professor of oral anatomy. This individual has been solely responsible for structure marking at our faculty for the past 15 years. This continuity ensures a highly standardized and precise marking procedure, minimizing inter-examiner variability and enhancing the consistency and reliability of the anatomical references used in the study. Detailed descriptions of the anatomical structures marked on each bone are provided in Table 1. Each student was instructed to examine and identify the marked structures on the bones independently, with a time limit of two minutes per specimen. During this time, students were permitted to handle the bone, rotate it, and inspect it from various angles to enhance their understanding and improve identification accuracy. However, students were cautioned not to touch the marked areas directly to prevent any displacement or loss of markers, which could potentially affect the assessments of subsequent participants. The examination process was consistently supervised by two of the study authors, who ensured that testing conditions remained standardized and that all procedures were followed accurately throughout the assessment period.

**Table 1:** Percentage of correct answers by bone and anatomical structure across student groups.

Bones and anatomical structures	Undergraduate students (2nd year) *N* = 55	Undergraduate students (5th year) *N* = 30	Postgraduate students *N* = 36	*P*-value
Mandible	95.5%	48.3%	52.8%	<0.001
Mental foramen	100%	90%	91.7%	0.031
Oblique line	98.2%	30%	50%	<0.001
Coronoid process	94.5%	73.3%	66.7%	<0.001
Masseteric tuberosity	89.1%	0%	2.8%	<0.001
*P*-value	0.031	<0.001	<0.001	
Sphenoid bone	93.6%	21.7%	11.8%	<0.001
Infratemporal crest	89.1%	3.3%	2.8%	<0.001
Pterygoid fossa	92.7%	16.7%	19.4%	<0.001
Hypophysial fossa	98.2%	60%	16.7%	<0.001
Foramen rotundum	94.5%	6.7%	8.3%	<0.001
*P*-value	0.288	<0.001	0.102	
Maxilla	91.4%	35.8%	37.5%	<0.001
Conchal crest	81.8%	10%	2.8%	<0.001
Incisive canal	98.2%	70%	69.4%	<0.001
Infraorbital foramen	94.5%	56.7%	63.9%	<0.001
Frontal process	90.9%	6.7%	13.9%	<0.001
*P*-value	0.024	<0.001	<0.001	
Temporal bone	96.8%	36.7%	32.6%	<0.001
Mastoid process	98.2%	50%	47.2%	<0.001
Mandibular fossa	92.7%	10%	13.9%	<0.001
Zygomatic process	100%	70%	58.3%	<0.001
Internal acoustic opening/meatus	96.4%	16.7%	11.1%	<0.001
*P*-value	0.219	<0.001	<0.001	
Cranium	85.9%	20.8%	25.7%	<0.001
Incisive foramen	100%	76.7%	77.8%	<0.001
Articular tubercle	85.5%	3.3%	19.4%	<0.001
Carotid canal	69.1%	3.3%	0%	<0.001
Hypoglossal canal	89.1%	0%	5.6%	<0.001
*P*-value	<0.001	<0.001	<0.001	

### Statistical analysis

Percentages of correct answers for different bones and anatomical structures were calculated for all three groups. To evaluate differences in the proportion of correct answers between groups, Fisher's exact test was applied. Additionally, *p*-values for differences in the percentage of correct answers across various anatomical structures within the same group were assessed (Table 1). The percentages of correct answers by bone were also evaluated across postgraduate groups, and the Kruskal–Wallis test was used to assess differences in years of experience across postgraduate groups (Table 2). *P*-values <0.05 were considered statistically significant.

**Table 2:** Percentage of correct answers by bone across different postgraduate student groups.

Skull Bones	Orthodontics *N* = 8	Endodontics *N* = 5	Prosthodontics *N* = 5	Periodontology *N* = 5	Maxillofacial radiology *N* = 3	Pediatric dentistry *N* = 6	Oral surgery and oral medicine *N* = 4	*P*-value
Years of experience as a dentist [Mean (SD)]	10.75 (3.99)	10.4 (5.94)	15.8 (4.44)	10 (3.39)	11.67 (7.23)	11.5 (7.15)	8 (0.82)	0.4548
Mandible	59.4%	35%	60%	35%	66.7%	41.7%	81.2%	0.037
Sphenoid bone	15.6%	15%	5%	5%	33.3%	0%	18.8%	0.048
Maxilla	28.1%	45%	40%	35%	50%	20.8%	62.5%	0.138
Temporal bone	28.1%	35%	40%	35%	16.7%	37.5%	31.2%	0.872
Cranium	12.5%	25%	30%	20%	41.7%	16.7%	56.2%	0.037
*P*-value	<0.001	0.296	0.004	0.083	0.165	0.002	0.003	

## Results

### Identification of anatomical structures across student groups

The percentage of correct answers for different bones and anatomical structures is summarized for second- and fifth-year undergraduate students, as well as postgraduate students (Table 1). The values in the rightmost column represent *p*-values comparing the performance of second- year students to that of fifth year and postgraduate students. Overall, second-year undergraduate students demonstrated significantly (*P* < 0.05) higher percentages of correct answers compared to fifth-year undergraduates and postgraduate students across all bones evaluated (Table 1). For second-year undergraduate students, the percentage of correct answers on individual bones was consistently high, exceeding 90% for each of the four single bones evaluated. Accuracy ranged from 91.4% for the maxilla to 96.8% for the temporal bone. However, when assessing the entire cranium, this group achieved 85.9% correct identification of anatomical structures (Table 1).

Significant differences in the percentage of correct answers for various anatomical structures within each bone group are indicated by the *p*-values listed below each group. For second-year undergraduate students, for three of the bone specimens, i.e., mandible, maxilla, and cranium, certain anatomical structures were more challenging to identify accurately than others (*P* < 0.05). The most difficult structure to identify on the mandible was the masseteric tuberosity (89.1%), the conchal crest on the maxilla (81.8%), and the carotid canal on the cranium (69.1%). In contrast, identification accuracy for specific structures on the sphenoid and temporal bones was more consistent, with scores ranging from 89.1% for the infratemporal crest to 98.2% for the hypophysial fossa on the sphenoid bone, and from 92.7% for the mandibular fossa to 100% for the zygomatic process on the temporal bone (Table 1).

A closer examination of correct scores among fifth-year undergraduate students revealed that the total percentage of correct answers across both individual bones and the entire cranium was below 50%, ranging from 20.8% for the cranium to a high of 48.3% for the mandible (Table 1). Additionally, the percentage of correct answers for various anatomical structures within each bone group varied significantly across all five specimens (*P* < 0.001). The greatest variation was observed in the mandible, where 0% of students correctly identified the masseteric tuberosity, while 90% correctly identified the mental foramen. In general, the results of fifth-year students were significantly lower than those of second-year students. However, certain anatomical structures were retained better by fifth-year students, such as the mental foramen (90% vs. 100%) and coronoid process (73.3% vs. 94.5%) on the mandible, hypophysial fossa (60% vs. 98.2%) on the sphenoid bone, incisive canal (70% vs. 98.2%) on the maxilla, zygomatic process (70% vs. 100%) on the temporal bone, and incisive foramen (76.7% vs. 100%) on the cranium. For the remaining anatomical structures, the discrepancy in correct identification percentages was substantial (Table 1).

The performance of postgraduate students closely resembled that of fifth-year undergraduate students. The highest total score for postgraduate students was observed for the mandible, with 52.8% correct identifications, while the lowest score was for the sphenoid bone, with only 11.8% correct identifications (Table 1). When examining the identification of individual anatomical structures on each specimen, postgraduate students performed similarly to fifth-year students, with a few exceptions, such as the oblique line on the mandible (50% vs. 30%) and the hypophysial fossa on the sphenoid bone (16.7% vs. 60%). Additionally, significant variation in the percentage of correct answers across different anatomical structures was found within four of the specimens (*P* < 0.001). The exception was the sphenoid bone, where most structures were poorly identified, resulting in no significant variation (*P* = 0.102) (Table 1).

### Correct answers by bone across different postgraduate student groups

The third group in the study consisted of 36 postgraduate students who were qualified dentists with varying levels of clinical experience and different intervals since graduation. This postgraduate group included representatives from seven specialist training disciplines: endodontics, prosthodontics, pediatric dentistry, periodontology, orthodontics, maxillofacial radiology, and oral surgery and oral medicine. The percentage of correct answers for each postgraduate group across different bone types is presented in Table 2, along with the average years of experience for students within each specialty.

Our results indicate no significant difference in years of experience among the different postgraduate groups (*P* = 0.45). However, significant differences were observed in the percentage of correctly identified structures among the specialist training disciplines for the mandible, sphenoid bone, and cranium (*P* < 0.05). Postgraduate students specializing in oral surgery and oral medicine achieved the highest accuracy for the mandible, with 81.2% correct identifications, while students in endodontics and prosthodontics had the lowest accuracy for this bone, with 35% correct identifications. For the sphenoid bone, maxillofacial radiology students performed best, with 33.3% correct identifications, whereas students in pediatric dentistry achieved 0% accuracy for this bone. In the case of the cranium, students specializing in oral surgery and oral medicine again performed best, with 56.2% correct identifications, while only 12.5% of orthodontics students correctly identified structures on this bone.

When examining differences in the percentage of correct answers across different bones within each postgraduate group, significant differences were found for the groups specializing in orthodontics, prosthodontics, pediatric dentistry, and oral surgery and oral medicine (Table 2).

## Discussion

Knowledge loss during the medical curriculum is a significant concern for both educators and students. Research shows that only about two-thirds of unrehearsed knowledge can be recalled after one year, and less than 50% after two years (4). The loss of knowledge that is neither used, revisited, nor relearned follows a negatively accelerated forgetting curve. However, retention eventually stabilizes at around 15%–20% approximately five years after learning has ceased, a phenomenon known as the “permastore” concept, where the remaining knowledge reaches a state of permanent storage (17). The retention of basic science knowledge, including anatomy, follows a similar trajectory (4). The primary findings from this study regarding fifth-year undergraduate students align with previous research, showing that less than 50% of osteology knowledge was retained after roughly two years, with some elements exhibiting even lower retention rates (Table 1). On the other hand, a more surprising finding was that postgraduate students, despite the passage of many years, retained osteology knowledge at similar levels to fifth-year undergraduate students.

To assess retention, fifth year undergraduates were given a selection of questions identical to those in the original preclinical examination of the year 2022. Given the 2.5-year interval and the unannounced nature of the re-examination, it is unlikely that students' scores were influenced by prior memorization of answers. In this examination, students were asked to complete 20 anatomical questions on 5 marked osteological specimens within a 10 min timeframe. Although this assessment may appear limited for evaluating the broad scope of head anatomy, it focuses on essential areas that were heavily emphasized during instruction in second year. As anticipated, second year undergraduate students achieved the highest scores (Table 1), following their recent completion of anatomy coursework and examination. The enhanced performance observed in this group of students can likely be attributed to the combined effects of impending examinations and intensive study sessions. This finding corroborates previous research that has shown that assessment-driven learning is associated with improved memory formation (18). Furthermore, it is well-documented that students' engagement and learning outcomes are profoundly influenced by their perceptions of the assessment methods used (19). Thus, the thorough processing of information prior to examinations explains the superior performance demonstrated by the second year undergraduate students.

In this study, specific osteological structures taught at the pre-clinical level, including those with clinical and radiographic relevance, were selected for assessment. This methodological choice enhances our examination of retention capabilities, supported by previous research, which suggests that information with significant practical relevance, such as clinically pertinent details, tends to be retained longer by students (20). Indeed, it is well-established that only information perceived as meaningful is committed to long-term memory (21). However, a detailed analysis of participants' performance revealed also unexpected patterns of misconceptions. Notably, there was frequent confusion between the masseteric tuberosity, a crucial point of insertion for the masseter muscle, and the angle of mandible. This confusion likely arises because the angle of mandible is a more palpable landmark during clinical examinations and is thus more familiar to practitioners. Studies suggest that in-depth processing of surface anatomy, as opposed to the origins and insertions of muscles, significantly enhances long-term retention of information (22). Another significant observation concerns the rapid deterioration of knowledge about the articular tubercle. Despite its pivotal role in understanding the mechanics of mastication and the physiology and pathology of the temporomandibular joint, both fifth year undergraduates and postgraduate students demonstrated a marked lack of retention regarding this structure. While the specific causes of this substantial knowledge decline remain elusive, our analysis revealed that, generally, more identification errors occurred across the entire cranium compared to individual bones in all groups studied, as indicated in Table 1. It is possible that this critical structure might be better recognized if explicitly highlighted on the temporal bone.

Moreover, it was apparent that anatomical features with direct clinical relevance exhibited considerably higher retention rates across both student groups. Notably, structures such as the mental foramen, coronoid process, incisive canal, infraorbital foramen, and incisive foramen demonstrated enhanced recall among participants. For example, the mental foramen was accurately identified by 90% of fifth-year undergraduate students and 91.7% of postgraduate students, as shown in Table 1. Furthermore, fifth-year students recognized the coronoid process with a 73.3% accuracy rate, whereas postgraduate students identified it at a rate of 66.7%, making the mandible the most frequently recalled bone. This finding is supported by postgraduate responses, especially from those specializing in oral surgery and oral medicine, where the mandible achieved the highest identification accuracy at 81.2% (Table 2). These observations are consistent with studies by Ghosh (2016) and Khin-Htun and Kushairi (2019), which suggest that the clinically engaged anatomy significantly boosts learning retention and knowledge preservation (23, 24).

Conversely, the results concerning the sphenoid bone reveal that both postgraduate and fifth-year undergraduate students struggled significantly with identifying its structures. Undergraduates achieved a 60% accuracy rate only for the hypophysial fossa, while other sphenoid structures had markedly lower correct identification rates ranging from 3.3% to 21.7%, as shown in Table 1. Among dental postgraduate students, those specializing in periodontology and prosthodontics demonstrated a particularly low accuracy rate of 5%, and pediatric dentistry residents were unable to correctly identify any structures on the sphenoid bone, as recorded in Table 2. The low identification rates among these specializations likely reflect the infrequent clinical use of these anatomical structures in their respective practices.

In the present study, the percentages of correct answers for various bones and anatomical structures were determined for each of the three groups. To assess differences in the proportion of correct answers between groups, Fisher's exact test was employed. Additionally, the *p*-values for differences in the percentage of correct answers across various anatomical structures within the same group were evaluated. Due to the small sample sizes in some groups, the exact test provides greater reliability than the more commonly used two-sample *z*-test, which assumes normally distributed proportions—a condition not reliably met with small samples. The percentages of correct answers by bone were also analyzed across postgraduate groups, and the Kruskal–Wallis test was applied to evaluate differences in years of experience among these groups. Given the somewhat limited sample sizes, a non-parametric test was deemed more reliable than ANOVA, as ANOVA assumes normally distributed averages, which may not hold true with small sample sizes.

A significant challenge and limitation in this study was the difficulty encountered in recruiting a larger number of fifth-year students. Despite participation being voluntary, 54.5% of eligible students took part in the examination. Recruiting students was particularly challenging due to their intensive curriculum and demanding class schedules at this stage of their education, further complicated by multiple exams taking place during the study period. A notable strength of the study, however, was our ability to longitudinally examine the same cohort of students at two distinct time points—during their second and fifth years of dental education, allowing for valuable insights into their retention of osteology knowledge. The recruitment of postgraduate students also presented several challenges, resulting in a limited sample size. Contributing factors included demanding class schedules, a high volume of patient treatments, smaller class sizes within each specialty, and some students being assigned to hospital duties, making them unavailable during the study period. Additionally, not all students were present at the time the study was conducted, which further affected participation. Consequently, the overall low and uneven distribution of participants in the postgraduate group presents a limitation and makes interpretation of the results more challenging. When considering all specialty groups together, the results presented in Table 1 are regarded as more reliable than when splitting the participants into subgroups by specialty (Table 2). However, due to the limited number of candidates currently enrolled at our faculty, it was not feasible to include more participants at this time. Future studies may benefit from focusing on including qualified specialists across various fields, as they may be easier to recruit and would likely provide comparable findings across specialties.

Although our results suggest that clinically relevant knowledge tends to be better retained over time, our findings clearly demonstrate, consistent with prior research (4), a significant decline in overall osteology knowledge as time progresses. This suggests that the clinical applicability of anatomical content plays a key role in how well it is remembered. It is reasonable to assume that anatomical landmarks frequently encountered in clinical practice, such as those visible on radiographs or used as surgical reference points, are reinforced through repeated exposure and application, leading to improved retention. In contrast, structures with limited direct clinical utility may be less frequently recalled and thus more susceptible to forgetting. These results underscore the importance of highlighting the clinical significance of anatomical features during teaching to support durable learning. Furthermore, they emphasize the need for curriculum design that aligns anatomical instruction with real-world clinical practice. By integrating such clinically important structures into both early and continuous phases of dental education, educators may enhance long-term retention and facilitate more effective clinical decision-making.

To support the consolidation of knowledge into long-term memory, additional cognitive processing is necessary following initial learning. This process is reinforced by the well-established “spacing effect,” which posits that information is better retained when it is reviewed at spaced intervals over time. Spaced learning can be implemented through the repetition of study material or, more effectively, through active retrieval via testing. Notably, empirical evidence suggests that repeated testing leads to superior long-term retention compared to repeated studying alone (25, 26). In the context of dental education, incorporating spaced retrieval practices, such as regular low-stakes quizzes, formative assessments, or cumulative review sessions, may significantly enhance the retention of osteological knowledge. This is particularly relevant for cranial bone anatomy, where a solid understanding of clinically important landmarks is essential for radiographic interpretation and surgical procedures. Additionally, continued testing post-graduation through clinical case discussions, licensing exam preparation, or continuing professional development courses may help sustain this anatomical knowledge throughout professional practice. By embedding spaced testing into both undergraduate education and lifelong learning, dental programs can better support durable knowledge of osteology, ultimately contributing to improved clinical competence and patient care.

The integration of anatomy into clinical education presents an ongoing challenge for many dental schools, largely due to the traditional separation between preclinical and clinical training phases (27, 28). In the early years of dental education, anatomy is often delivered through isolated, department-led courses (28), while in later stages, it frequently competes for curricular time with clinical training and other subjects. This division limits opportunities for meaningful reinforcement of anatomical knowledge in clinical contexts (24, 29). Our study highlights the potential benefits of greater vertical integration, a concept increasingly recognized as a valuable goal in medical and dental education (26). Vertical integration, where anatomy and clinical sciences are taught concurrently across the educational continuum, has been shown to enhance long-term anatomical recall (30) and help bridge the gap between theoretical knowledge and clinical application. While early curricula may successfully incorporate clinical topics (11), the integration of foundational subjects such as anatomy in later years remains a significant challenge. Nonetheless, early exposure to clinical settings has been associated with improved understanding of anatomy's relevance, enhanced student attitudes, and faster development of clinical competencies (31, 32). Presenting anatomical concepts within a clinical framework fosters a more cohesive understanding of pathology and diagnosis, supporting both retention and practical application (33). Furthermore, initial clinical experiences can reignite students' interest in dentistry, particularly during phases dominated by theoretical instruction. For this reason, increasing patient contact in the first year of dental education has been recommended to support engagement and learning (34). Concludingly, the vertical integration offers a viable strategy for maintaining the relevance of anatomy throughout the undergraduate years and better aligning basic science instruction with clinical training goals (35).

## Conclusions and future perspectives

Integrating clinical, radiological, and surface anatomy into continuing education programs for dental professionals may significantly enhance knowledge retention. Our study demonstrates rapid declines in osteological knowledge, underscoring the urgency for interventions to improve long-term retention early in educational and clinical training. The implementation of spaced learning and testing through universally accessible digital platforms could systematically improve the retention of anatomical knowledge across undergraduate education and clinical dental practice. Consequently, the development of such digital platforms, extending from osteology to other areas of clinical anatomy for dentists, and their impact on learning retention, warrants further studies and evaluation.

## Data Availability

The original contributions presented in the study are included in the article/Supplementary Material, further inquiries can be directed to the corresponding author.
